# Rapid spread of a novel NDM-producing clone of *Klebsiella pneumoniae* CC147, Northern Italy, February to August 2023

**DOI:** 10.2807/1560-7917.ES.2023.28.42.2300522

**Published:** 2023-10-19

**Authors:** Irene Mileto, Greta Petazzoni, Marta Corbella, Stefano Gaiarsa, Cristina Merla, Angela Kuka, Marina Ramus, Cristina Terulla, Micaela Brandolini, Antonio Piralla, Patrizia Cambieri, Fausto Baldanti

**Affiliations:** 1 Microbiology and Virology Unit, IRCCS Fondazione Policlinico San Matteo, Pavia, Italy; 2 Specialization School of Microbiology and Virology, University of Pavia, Pavia, Italy; 3 Department of Clinical, Surgical, Diagnostic and Paediatric Sciences, University of Pavia, Pavia, Italy; 4 ASST Pavia, Ospedale Civile di Voghera, Voghera, Italy; 5 ASST Pavia, Ospedale Unificato Broni-Stradella, Internal Medicine Unit, Stradella, Italy; * These authors have contributed equally; ** These authors contributed equally to the work and share the last authorship

**Keywords:** epidemiology, public health, surveillance, genomics, NDM-producing, Klebsiella pneumoniae

## Abstract

New Delhi metallo-beta-lactamase (NDM)-producing *Klebsiella pneumoniae* (*Kp*) ST147 caused a large multi-hospital outbreak in Italy from 2018 to 2021. We describe a new ST6668 NDM-producing *Kp* clone, belonging to CC147, which rapidly spread across hospitals in the Pavia province (Northern Italy) from February to August 2023. Genomic analyses revealed that ST6668 is different from ST147 and fast evolving. As shown here, genomic surveillance programmes are useful for tracking the spread of new clones with reduced susceptibility to most antibiotics.

The spread of New Delhi metallo-beta-lactamase (NDM)-producing *Klebsiella pneumoniae* (*Kp*) is of potential public health concern because these strains are transmitted in healthcare settings and are resistant to nearly all antibiotics. Cefiderocol or ceftazidime-avibactam in combination with aztreonam are the preferred antibiotic options for NDM-producing Enterobacterales [1].

Several sequence types (ST) of *Kp* worldwide, such as ST11 and ST147, have been reported to produce NDM carbapenemase. In particular, ST147 (and the clonal complex (CC) associated to it) is a high-risk international clone [2] that has caused a large outbreak in Italy (Tuscany region) in the period 2018 to 2021 [3,4]. Here we describe the isolation of a new ST of NDM-producing *Kp* (ST6668) belonging to CC147 that caused a multi-hospital outbreak from February to August 2023 in the Pavia province (Northern Italy).

## Event description

**Figure 1:**
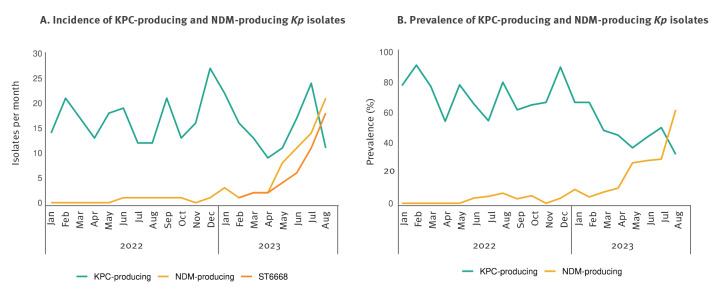
KPC and NDM-producing *Klebsiella pneumoniae* isolates, Fondazione IRCCS Policlinico San Matteo, Pavia, Northern Italy, January 2022–August 2023 (n = 394)

Until 2022, *K. pneumoniae* carbapenemase (KPC) has been the most detected carbapenemase in *Kp* at Fondazione IRCCS Policlinico San Matteo in Pavia (OSM), a 900-bed teaching hospital. The KPC incidence remained constant from August 2022 to August 2023 (16.3 ± 5.6 isolates/month), while the NDM incidence increased from April 2023 (Figure 1A). Indeed, among the carbapenem-resistant *Kp* isolates, the NDM prevalence reached 61.8% in August from 10% in April 2023 (Figure 1B).

*Kp*: *Klebsiella pneumoniae;* KPC: *Kp* carbapenemase; NDM: New Delhi metallo-beta-lactamase.

From January to August 2023, 117 isolates of NDM-producing *Kp* were collected at OSM from 66 patients. Considering the first isolate of NDM-producing *Kp* for each patient, we performed whole genome sequencing of 66 isolates, using Illumina reads as in [5]. In addition, we sequenced the genomes of eight NDM-producing *Kp* isolated from patients admitted to Ospedale Civile di Voghera, a 200-bed hospital in the Pavia province (ca 30 km from Pavia), belonging to the Pavia Health Authority Unit (Pavia-HAU). Genomic analysis of all 74 isolates showed that 52 of them belonged to the same clone, which presented a novel MLST allelic profile. The profile was submitted to the BIGSdb service (https://bigsdb.pasteur.fr) and confirmed as a new clone, named ST6668. This new clone has since February 2023 been responsible for the rapid rise in NDM-producing *Kp* as shown in Figure 1A *.*

Of the 52 *Kp* ST6668 strains, 44 had been collected from patients in OSM and eight in Voghera hospital. Forty-four of them were isolated from rectal swabs and urine samples, five from blood cultures, and the remaining three from bronchoalveolar lavage, pus and drainage liquid. The patients had a mean age of 70 years (SD: ± 15.4), ranging from 31 to 90 years, and 31 of the 52 were male. At OSM, 13 patients were admitted to specialist medical care (pneumology, nephrology/rheumatology and infectious diseases), 10 to oncology wards, seven to intensive care units, five to surgery wards, three to general medicine, while the remaining six were outpatients. At OSM, 42 patients were screened for multidrug-resistant (MDR) Enterobacterales colonisation via rectal swab, as foreseen by internal procedures for high-risk patients and those hospitalised on critical wards. Of these, 36 were colonised at admission, while the remaining six became colonised during hospitalisation (mean value: 15 days after admission). Seven patients developed bloodstream or pulmonary infections. Moreover, three of the eight Voghera patients were later transferred to a small hospital of the Pavia-HAU (20 km from Pavia and Voghera).

## Clone characterisation

Antimicrobial susceptibility testing was performed using Sensititre DKMGN broth microdilution (ThermoFisher) and interpreted according to European Committee on Antimicrobial Susceptibility Testing (EUCAST) clinical breakpoints [6]. All 52 isolates were resistant to most antibiotics including carbapenems, ceftazidime-avibactam and amikacin, while only two were resistant to cefiderocol. All isolates remained susceptible to gentamicin. The minimal inhibitory concentrations (MIC) are listed in the Supplementary Table. The combination of ceftazidime-avibactam with aztreonam had a synergistic effect on all the strains [7]. 

**Figure 2:**
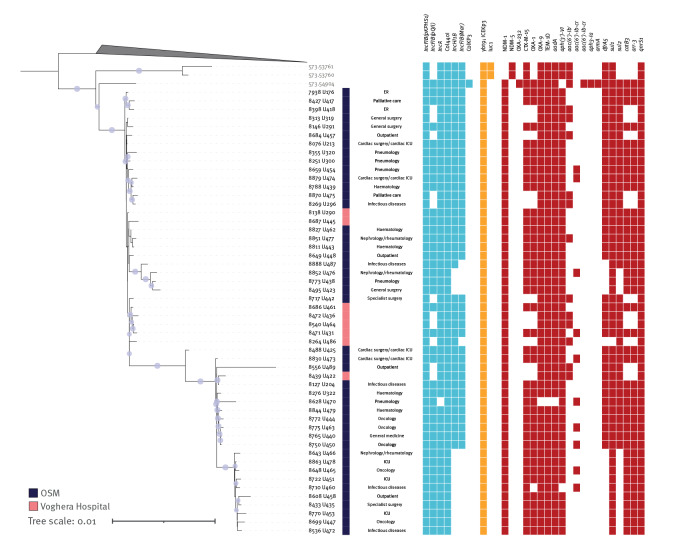
Phylogenetic analysis of carbapenemase-producing *Klebsiella pneumoniae* ST6668 isolated at two hospitals, Northern Italy, February–August 2023 (n = 52)

ER: emergency room; ICU: intensive care unit; *Kp*: *Klebsiella pneumoniae*; OSM: Fondazione IRCCS Policlinico San Matteo.

Illumina short reads were de novo assembled with Shovill (github.com/tseemann/shovill). We used P-DOR [8] to obtain the single nucleotide polymorphism (SNP)-based maximum-likelihood phylogeny of the 52 isolates described in this work and the 38 most similar high-quality genomes available from the BV-BRC database (bv-brc.org) (Figure 2). The 52 genomes from the Pavia area resulted to be monophyletic (SNP range: 4–51), suggesting that all strains were part of the same epidemic event. All outbreak isolates belonged to ST6668, which differs from ST147 by a point mutation (C414T) in the *phoE* locus; whereas the 38 genomes used as background belonged to ST147. The three closest genomes to the outbreak clade were isolated in Lebanon from blood (in 2020) and in the United States from urine and rectal swabs (in 2022). Moreover, none of the closest strains retrieved in the phylogeny was the ST147 clone responsible for the large outbreak in Tuscany [4]. This suggests that the ST6668 clone originated from a separate importation of CC147.

Maximum-likelihood phylogeny of 90 *Kp* strains inferred on coreSNPs with RAxML, including 52 from the outbreak (black labels) and 38 from the BV-BRC database (grey labels – 35 leaves have been collapsed). Bootstrap values above 70/100 are indicated with violet dots. Isolate information is reported alongside the tree, including the hospital of isolation (OSM/Voghera hospital). The OSM wards are also indicated. Presence/absence matrix shows the pattern of plasmids (blue), virulence determinants (yellow) and resistance determinants (red).

We used Kleborate [9] to search for determinants of antibiotic resistance and virulence, and PlasmidFinder [10] to determine the plasmid presence/absence profile. The Supplementary Table add information on genomic resistance determinants, virulence factors and plasmids presence. All outbreak strains harboured the *bla_NDM-1_* gene conferring carbapenem resistance. In addition, all strains presented allele 9 of the *ybt* gene, encoding yersiniabactin, enclosed in the ICE*Kp*3 integrative island. Six plasmid sequences were detected in the outbreak. Two of them were common to all 52 isolates (*IncFIB(pKPHS1)*, *Col440I*), while the other four presented a presence/absence pattern, which has a high level of concordance with that of antimicrobial resistance determinants (Figure 2). In particular, *bla*_CTX-M-15_, *bla*_OXA-1_, *catB3* and *arr-3* were associated with plasmid *IncFIB(pQil)*, while *dfrA5* and *sul2* were associated with plasmid sequences *IncHI1B* and *IncFIB(Mar)*. This result suggests a scenario of frequent exchange of gene content. The hypothesis is reinforced by the phylogeny: isolates that have lost plasmids *IncHI1B* and *IncFIB(Mar)* (and the associated resistance genes) are clustered into two high confidence monophyletic subclades (Figure 2).

## Discussion

Here we report the isolation of a new *Kp* clone, ST6668, which has spread among a considerable number of patients of two hospitals in the Pavia province within few months. The new clone was isolated from 46 inpatients and six outpatients, suggesting that it is presently circulating also beyond the healthcare setting.

Phylogenetic analysis of the 52 epidemic isolates showed that the cluster of ST6668 genomes presents a few high confidence monophyletic subclades, even though internally not fully resolved. The eight isolates from Voghera hospital did not cluster in one single subclade, suggesting multiple exchange events between hospitals in the Pavia area.

The emergence of the new clone has caused a shift in the epidemiology of carbapenemases at OSM, where KPC had been the prevalent carbapenemase. Notably, NDM detection has increased since April 2023, becoming the most detected carbapenemase at OSM in August 2023.

The presence in the new clone of a large number of resistance genes, including *bla_NDM-1_*, could have facilitated the rapid spread of this clone [11], while no particular virulence factor was observed. Moreover, the high mean age of the patients involved suggests that they may have a history of frequent admissions to hospitals or long-term care facilities. This could have favoured the spread of the ST6668 over the Pavia area. Indeed, three patients were transferred from Voghera to another nearby hospital, belonging to the Pavia-HAU. This scenario underlines the need to establish local, regional and international surveillance and infection prevention strategies [12] in any healthcare setting. In particular, real-time genomic surveillance has proven essential in the timely detection of novel fast-spreading clones and variants of clinical importance [13,14]. 

Although most of the patients of this study were colonised by ST6668 *Kp* without developing infections, hospitalised patients tend to remain colonised with MDR strains for the duration of their stay, increasing the risk of transmission of MDR strains to other patients [15]. 

## Conclusion

The fast spread of ST6668 and other NDM-producing clones should be controlled, since their reduced susceptibility to most antibiotics allows few therapeutic options. In light of how rapidly the new clone has spread within the healthcare setting in the Pavia area, we underline the importance of prompt communication between microbiologists and clinicians.

## Supplementary Data

320000 Supplement
